# Extract of a Lecture on Syphilis

**Published:** 1843-08

**Authors:** Philip Ricord


*Extract of a Lecture on Syphilis.*—By Dr. Philip Ricord. It is, gentlemen, to meet some of the difficulties previously mentioned that I have determined, upon upon mature reflection, to dedicate one entire lecture to the most proper mode of investigating a case of syphilis, and to demonstrate to you the degree of confidence which may be placed in the assertions of patients.

But, before I enter upon this subject, I beg distinctly to state my most unqualified respect for those convictions, without which the very existence of constituted societies is at stake. For female virtue—that guardian of the honor of men —I have the deepest admiration; but recollect, gentlemen, it is not with homilies on chastity that a course of syphilis can be carried on; and although I conceive it to be obligatory on the physician in practice not to destroy those illusions on which, occasionally, the peace of a family may depend, but to palliate the painful truths which might cause the annihilation of individuals, yet I conceive it to be my duty, in this amphitheatre, to raise the curtain, because science must not be made to consist of the delusions of love-sick youths, but of all the elements of truth which can be collected by manly and searching intellects.

Every day, gentlemen, persons affected with syphilis deny the possibility of the fact, from their knowledge of the "respectability" of the lady who has honored them with her favours. Let us, therefore, examine what conditions can place a woman above suspicion.

Is it the fact of her being free from the sacred bonds of matrimony? This question I shall answer with an anecdote borrowed from Dupuytren's practice. That great surgeon performed suture on a lady in whom the perineum had been torn during parturition. A couple of years afterwards a friend and patient requested the professor to visit his young wife, with whom the happy bridegroom had been unable to effect complete intercourse. Dupuytren, to his great surprise, recognised in the "too perfect" maid the lady whom he had previously attended, and found he had been himself the involuntary cause of the deluded husband's ill success; the sutures had, in fact, caused the partial obliteration of the genital organs. He remedied the injury, and, of course, was discreet.

In consequence of the brutal and stupid superstition prevalent amongst the lower orders, that connection with a "real maiden" is the only effectual way of removing inconvenient syphilitic symptoms, I have myself seen a little girl, not eighteen months old, with chancres and buboes.

A man servant called on me not long since, laboring under gonorrhœa. His first statement was, that six months had elapsed since the last sexual intercourse. On being hard pressed with questions, he admitted that, being about to be married (two banns had been already proclaimed), he had considered himself justified in levying a tythe on his future rights. I examined the "blushing bride," and after much difficulty, she acknowledged that violence had been lately used towards her by another person; but, added she, to a certainty he was not diseased, for if he had been, she would positively have resented his rudeness by "raising the house." I need not tell you, Gentlemen, the third bann never was published.

Two physicians, beside myself, attended a young gentleman with constitutional syphilis. He mentioned having had intercourse with two women of suspicious character, whom the medical attendants examined separately, and found healthy. A month elapsed, and I at last discovered the real cause of this gentleman's amorous misfortunes to lie with a *married* lady of unblemished repute and high station, who had sent for me, not in my medical capacity, for she did not imagine herself infected, but to secure my attentions for the young gentleman above mentioned, whose disorderly conduct gave her no small uneasiness.

Who has not heard the famous history of Sidrac, who was presented with chancres in return for his first visit to the connubial bed. "It is true," says Voltaire, "it was a family complaint."

I must say, gentlemen, it is my conviction, more syphilis has its origin beyond the pale of prostitution than within it. But, even in Paris, what degree of security can public health derive from the medical examination of prostitutes once a week, or of unlicensed women once a fortnight, when we know *three days* to be amply sufficient for a sore to be communicated, and rendered inoculable, when we know from *our own* experience that infected women send to the physician in their stead one who does not fear inspection, and who thus brings home to them a stamped and signed permission to inflict on the unguarded and the imprudent the most horrible disease with which Providence has been pleased to chastise mankind!

The "profession" of syphilitic patients is occasionally brought forward as an unanswerable proof of the spontaneous development of the disease, or at least as sufficient evidence of contamination, without the participation of the patient. Now, notwithstanding my profound and sincere respect for religious institutions, I conceive it would be offering an insult to your better judgment, were I to propose to you seriously, as Fallopia does in a satirical mood, in explanation of venereal symptoms observed in a community of nuns, "the promiscuous use of holy water!"

My colleague, Dr. Serres, one of the most learned and distinguished members of the Academy of Sciences, Paris, was called in to attend a young lady who was troubled with hæmorrhoids. These turned out to be *bona fide* "chancres." The Doctor could not credit the evidence of his senses; he held a private conference with the young lady's mother, and, to his utter dismay and confusion, found her infected also, but in a manner more consonant with general custom. Further investigation showed that a *reverend* adviser of this interesting family had extended his attentions to both mother and daughter, and had left after him most painful marks of his kindness.

In the investigation of patients, physicians are often exposed to infection. I will select one out of many well authenticated instances. Our late lamented colleague, Dr. Hourmann, of the Hopital de l'Ourcine, in examining a syphilitic woman, inoculated a chancre on his finger. Secondary and tertiary symptoms were the consequence, and after ten months the disease terminated fatally.

This, however, is far from being the most frequent mode in which professional persons are diseased. A midwife with a papular and scaly eruption, was lately considered by Dr. Casenave as an instance of infection by *simple absorption* from the finger; she, of course encouraged the belief. On being cross-examined by myself, she acknowledged, with tears, that one single, never-since-repeated breach of her marriage articles had been visited on her thus severely, and that she would take good care to be perfectly faithful for the future to her lord and master.

When all these circumstances are reflected upon—when it is recollected that illicit and disgusting practices, too common in highly civilized countries, often produce primary sores of the anus, mouth, &c.—that a sense of shame will compel such patients to conceal the disgraceful truth, unless the avowal be actually wrung from them by the fear of consequences—can it be wondered at if the true origin of many cases of syphilis remains shrouded in doubt?—but is that origin, therefore, to be denied?

A class of females to which we are all, more or less, indebted—nurses—often lay upon their unconscious suckling the blame which more properly rests with older offenders. I have had under my care an infected nurse, who threw the entire onus upon an innocent baby, although she had taken, at the same time, "to nurse," an unlimited number of bold dragoons. A few days since a patient left our wards, cured of constitutional symptoms; his wife had communicated to him an indurated chancre some five months before. Here, again, a child she had nursed three years previously was made the scapegoat, and the poor husband never once reflected he had been living in the dangerous vicinity of a garrisoned town.

In the examination of syphilitic patients, we must not, gentlemen, lose sight of the disease previous to the present one—of the period at which the present symptoms may have arrived—nor of the possibility of total disppearance of former accidents. You must not forget that a phymosis may conceal the primary sores; that they may lurk within the folds of the vagina, or even be engrafted on the os uteri itself. This last remark leads us to our concluding observation, that all syphilitic cases in women, where the use of the speculum has been neglected, shall be for us as though they never were recorded.—*Medical Examiner*, from *Prov. Med. Journ.*

